# Prosthodontic Rehabilitation With Kennedy’s Class I and Class II Using an Extended Precision Attachment: A Report of Two Cases

**DOI:** 10.7759/cureus.43723

**Published:** 2023-08-18

**Authors:** Tushar S, Ananya Singh, Priya Rani, Jayant Prakash, Sunila BS, Shivakumar G C

**Affiliations:** 1 Department of Prosthodontics, Dental Institute, Rajendra Institute of Medical Sciences, Ranchi, IND; 2 Department of Dentistry, Sadar Hospital, Muzaffarpur, IND; 3 Department of Prosthodontics and Crown and Bridge, Jagadguru Sri Shivarathreeshwara (JSS) Dental College and Hospital, JSS Academy of Higher Education and Research, Mysore, IND; 4 Department of Oral Medicine and Radiology, People’s College of Dental Science and Research Center, Bhopal, IND

**Keywords:** kennedy’s class i and ii, metal framework, extracoronal attachment, precision attachment, rhein 83

## Abstract

Satisfactory restorations can be difficult in partially edentulous patients, especially those with unilateral or bilateral posterior ocular defects. With traditional and modern treatment options, recovery can be successful. Partial dentures with attachments are such a treatment. An implant-supported prosthesis is another option for therapy in these circumstances. Precision extracoronary attachments are the preferred treatment option when implant treatment does not give good results. This research offers two examples of partial cast prosthetic rehabilitation for distal extension utilizing precise attachments.

## Introduction

Successful prosthetic restorations, both aesthetically and functionally, require meticulous care and careful treatment planning. The treatment options available to replace partially missing teeth include fixed partial dentures, partial dentures that are detachable and have clasps, removable partial dentures (RPDs) with precision attachment, implants, and overdentures. Fixed restorations are undoubtedly the universal answer to most restorative alternatives because they are clean, aesthetically pleasing, and preferred by most patients, but when the remaining ridge structure is lost vertically, their application is not universal because of the limitations and the scope of remediation, and more assistance is needed. The complexity and the cost of prostheses are important issues to consider [1]. When there is not enough bone mass and when there are financial constraints, implant-supported dentures cannot be planned. Therefore, acrylic partials or cast partials should be preferred in such situations. The primary goal when replacing missing teeth is to restore function and preserve oral tissue and aesthetics. Ignoring these factors and focusing solely on replacing missing teeth, many removable partial dentures (RPDs) are manufactured. This negatively affects the remaining natural dentition. Distal extension rehabilitation with prosthetics in the partly edentulous condition is still difficult. The distal abutment and the edentulous ridge provide support for the distal extension RPD. The terminal abutment acts as a fulcrum, and the functional movement of the RPD and retentive clasp can apply forces to the abutment and damage the periodontal tissue of the abutment [2].

Because they include clasps and other precise attachment components, cast partial dentures are retentive. An attachment is described as “a mechanical device for fixation, retention, and stabilization of a prosthesis” [3]. This is a two-part connector: one part is fixed to a tooth, implant, or root, and the other is connected to the prosthesis.

Mensor [4] identified the types of attachments as auxiliary, push-button type, bar type, intracoronal, and extracoronal. Depending on their resilience, they may also be categorized as solid or stiff, with and without a screw or U-pin (hinge, rotatory type, and vertical). It is actually recommended to use resilient kinds to prevent torque on the abutment tooth in the instance of a prosthesis supported by tooth tissue [5]. Function, location, space, retention, and cost-effectiveness are the main selection factors. Depending on the location, extracoronal, intracoronal, root/intra-root pin type, and bar type may be utilized. Dr. Herman Chayes initially described the intracoronal type in 1906, and it consists of a male flange and a female slot (female) [6]. Intracoronary attachment occurs inside the cusps, the usual proximal axial contour, or the typical crown contour. Extracoronal refers to an attachment that is placed outside the native tooth’s crown. A firm or flexible connection between the tooth and prosthesis is made through extracoronal attachments, which protrude from the crown. Studies have shown that the survival rates are 83.35% in five years, 67.35% in 15 years, and 50% in 20 years [7]. This paper has outlined two situations: a modification I to Kennedy’s class II and bilateral distal extension. Two prosthetically restored cases with extracoronal castable precision attachments make up Kennedy’s class I cases.

## Case presentation

Case 1

**Figure 1:**
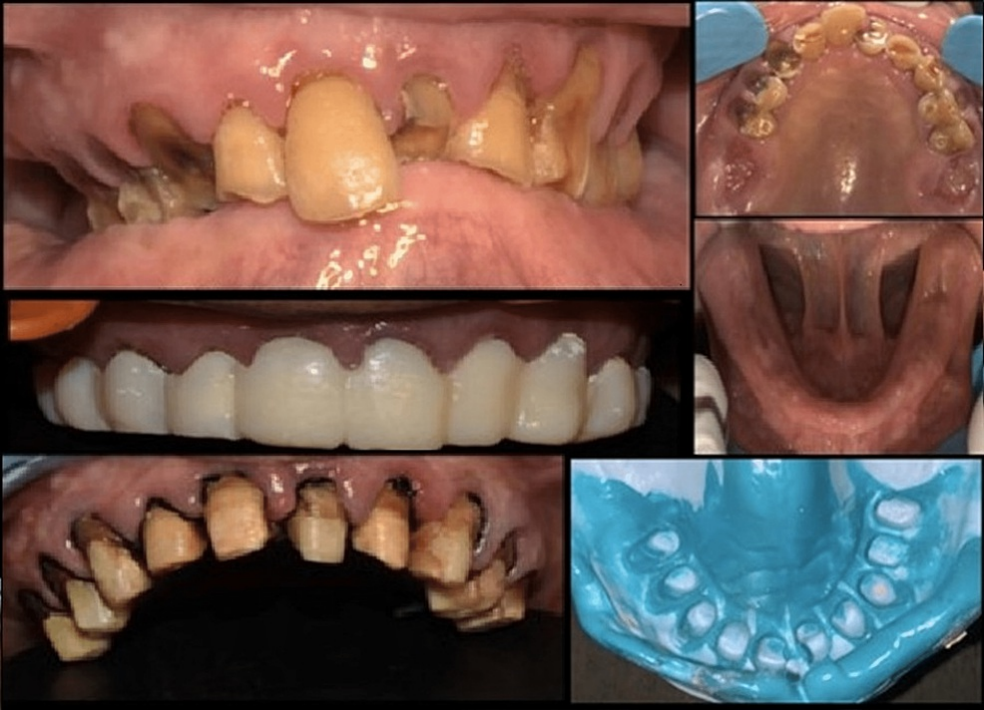
Intraoral images with tooth preparation with temporization and final impression

A 60-year-old male presented with missing upper molars and poorly fitting acrylic partial dentures. Intraoral examination revealed bilateral upper molars (Kennedy’s class I) and a completely edentulous mandibular arch. While the remaining maxillary teeth were periodontally stable, they required post and core restoration after root canal treatment. A comprehensive clinical and radiological assessment was performed to develop a prosthetic treatment plan. The plan involved composite prostheses with extracoronal precision attachments on both maxillary distal extension arches and complete prostheses on the mandibular arch. After root canal treatment, post and core tooth preparation was carried out for all remaining teeth. Abutments 11, 21, 12, 22, 13, 23, 14, 15, 24, and 25 were used to obtain metal-bonded porcelain crowns. Provisional restorations were placed on the prepared abutments after the final impression was taken (Figure 1).

Laboratory Procedure

Abutments 11, 21, 12, 22, 13, 23, 14, 15, 24, and 25 were waxed up, and the lingual region of the metal-ceramic abutments was polished. The best attachment site was determined using the appropriate parallelometer mandrel, joint space, and bulk assessments.

Casting Trial for Porcelain-Fused-to-Metal (PFM) Crowns With Attachments and Metal Framework

**Figure 2:**
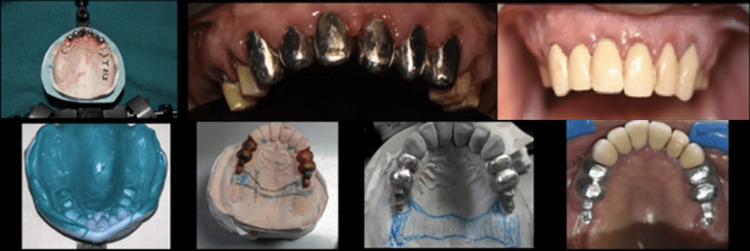
Fabrication of the maxillary prosthesis

Metal-ceramic crowns with attachment structures were cast and porcelain-fired. Single crowns for 11, 12, 13, 23, 21, and 22 and joint crowns with attachments were fabricated in the laboratory and tested for exact fit. Cast partial dentures with attachments were manufactured in the laboratory and trialed on metal frameworks in the patient’s mouth. The jaw connection was documented, and the stability and correctness of the casting framework were examined (Figure 2).

Wax-Up Trial

**Figure 3:**
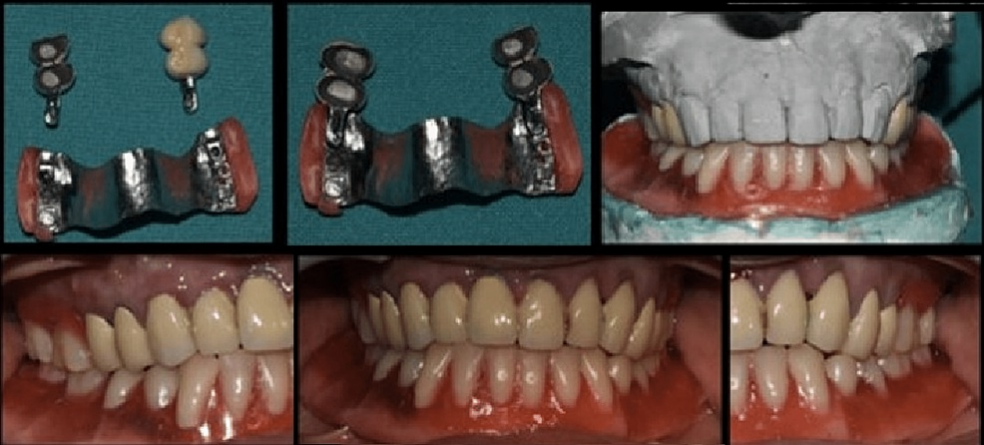
Trial of maxillary and mandibular framework

Teeth were waxed in the patient’s mouth, and a teeth-setting experiment was conducted. After the denture trial was submitted for acrylization, a cast partial denture was completed (Figure 3).

Placement of the Combined Prosthesis in the Patient’s Mouth

**Figure 4:**
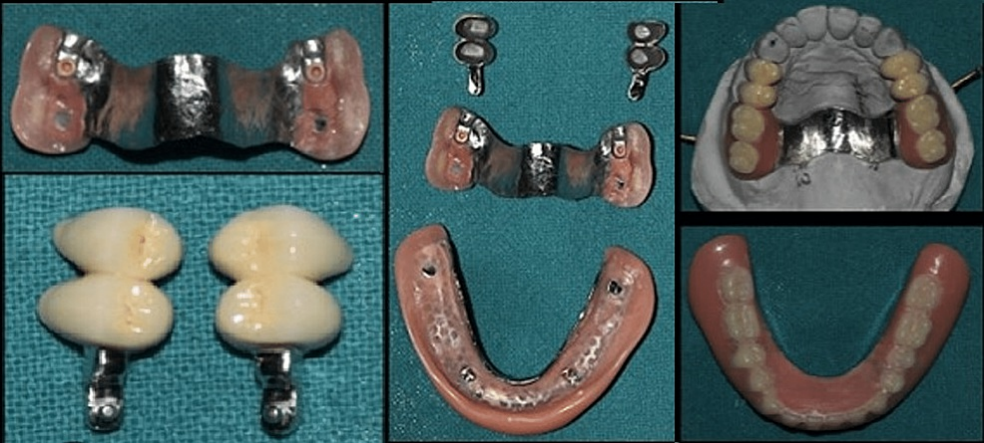
Final prosthesis

The finished prosthesis was trial-seated, and the crowns were attached using glass ionomer cement (GC Fuji, GC Corporation, Tokyo, Japan). A small layer of Vaseline was applied to the attachments to facilitate the removal of the cast partial denture. The mandibular full denture was also placed in the patient’s mouth along with the final maxillary combination prosthesis using an extracoronal castable distal extension precision attachment. A follow-up call was made to the patient after 24 hours for post-insertion assessment (Figure 4).

Case 2

**Figure 5:**
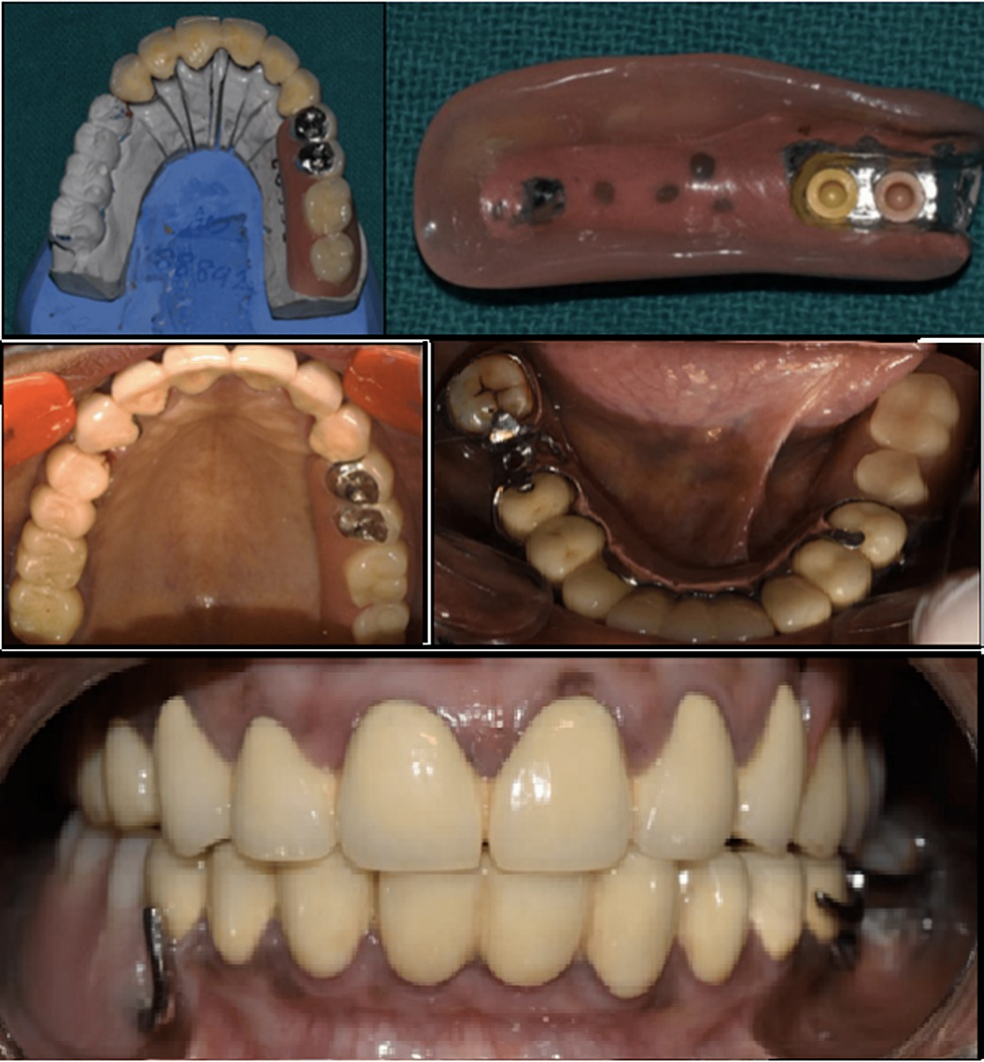
Complete procedure

A 54-year-old female with missing maxillary posterior teeth and a mandibular Kennedy’s class II modification I presented to the department. All the teeth were periodontally stable but exhibited severe attrition, requiring root canal treatment followed by PFM crowns with precision attachment in the maxillary region and PFM crowns and cast partial denture in the mandibular region (Figure 5).

## Discussion

Precision attachment refers to a connection involving two or more components. It entails mechanically joining one part to the implant, tooth root, or prosthesis, while the other part is attached to the prosthetic. These attachments allow for the combination of benefits from fixed teeth and removable dentures, presenting a versatile solution. Notably, in the early 20th century, Dr. Herman Chayes was the pioneer in describing the formation of attachments.

With precise attachments, removable prostheses offer numerous advantages, including improved aesthetics, fewer postoperative adjustments, and increased comfort. These benefits are particularly relevant for wide edentulous jaws, nonparallel abutments, and distal extension bases [8]. Numerous studies have shown that attachment-supported cast partials offer superior function, aesthetics, and comfort while also protecting the abutments and requiring fewer adjustments. Moreover, patients frequently prefer and find them easier to clean and wear. The utilization of these attachments in fixed prostheses, implant treatments, and overdentures significantly contributes to prosthesis success in terms of aesthetics, comfort, and functionality [9].

Intracoronal attachments, on the other hand, necessitate more tooth reduction and weaken tooth structure due to their higher vertical height requirements to fit the connection. As a solution, an additional coronal attachment is applied. Specifically, the extracoronal castable RHEIN 83 OT CAP attachment system, utilized in the cases analyzed in this paper, is positioned distally to the crown as an extension, providing ample vertical space for optimal aesthetics. Shaping the castable OT CAP male alongside the crowns during the waxing-up stage avoids difficult adaptation operations, such as metal welding connection after crown casting. The female component is equipped with a retention nylon cap color-coded according to retention properties, while the male component features a flat head ball design [10].

An essential factor for the effectiveness of a distal extension cast partial denture is stress management on the abutments. This is achieved through a dual impression process, wide coverage, a secure denture base, stiff design, physiologic shimming, and splinting of the abutments, all combined with appropriate attachments. The proposed method proves superior to the traditional prosthesis in several aspects. In the case studies presented, the abutments’ crown height was sufficient to support the attachment, and many abutments were splinted in front of the edentulous span to evenly distribute stresses. Additionally, maintenance of oral hygiene is significantly simplified with a cast partial denture due to its detachable nature. Moreover, the treatment is more cost-effective, and laboratory procedures are streamlined with readily accessible preformed prosthetic components. Consequently, attachment-retained partials offer patients greater long-term security and satisfaction compared to clasp-retained partial dentures [11].

## Conclusions

Removable partial dentures remain a viable therapeutic option for individuals with class I and class II Kennedy’s partial edentulous disorders. To enhance the retention, appearance, and functionality of these partial dentures, precision attachments such as the RHEIN 83 OT CAP attachment system can be employed, given appropriate case selection and treatment planning. Utilizing the aforementioned technique, we can offer patients a highly functional and comfortable prosthetic option, facilitating the bilateral and unilateral distal extension of the edentulous jaw. By simply inserting a retention cap into the prosthesis framework, attachment retention can be easily monitored and improved, leading to enhanced patient comfort and satisfaction.
